# Apical Extrusion of Debris after Canal Preparation with Hand-Files Used Manually or Installed on Reciprocating Air-Driven Handpiece in Straight and Curved Canals

**DOI:** 10.7508/iej.2015.03.004

**Published:** 2015-07-01

**Authors:** Hossein Labbaf, Leila Shakeri, Reza Orduie, Farshid Bastami

**Affiliations:** a *Endodontic Department, Dental School, Shahed University, Tehran, Iran; *; b * Private Practice, Tehran, Iran; *; c *Dental School, Shahed University, Tehran, Iran; *; d *Research Institute of Dental Sciences, Shahid Beheshti University of Medical Sciences, Tehran, Iran*

**Keywords:** Apical, Apical Extrusion, Debris Extrusion, NiTi Files, Reciprocation, Step-Back Technique

## Abstract

**Introduction:**

Apical debris extrusion (DE) subsequent to root canal instrumentation, is one of the most important causes of endodontic flare-ups. The aim of this study was to compare the amount of DE after root canal instrumentation using nickel-titanium (NiTi) hand files with step-back manual technique or installed on reciprocating handpiece.

**Methods and Materials:**

This study was conducted on mesiobuccal (MB) roots of extracted maxillary first molars (*n*=20) and roots of mandibular premolars (*n*=20) that were randomly divided into two groups (*n*=20) according to the armamentarium used for canal preparation (air-driven reciprocating handpiece or hand instrumentation). In each group, the MB and premolar roots were prepared with the main apical sizes of 35 and 40, respectively. The extruded debris were collected and weighed. Finally, the mean dry weights were compared using ANOVA and t-test, and Tukey’s Multiple Comparisons Procedures were used to determine the significant differences in amounts of DE. The level of significance was set at 0.05.

**Results:**

Regardless of the type of teeth, the mean values of DE, were significantly lower in the handpiece group (*P*<0.0001). In addition, significantly lower amounts of DE was observed in premolars in similar group (*P*<0.001). However, this difference was not significant in MB roots of molars (*P*=0.20).

**Conclusion:**

Root canal preparation with reciprocating handpiece can lead to significantly lower debris extrusion than the manual step-back technique. In handpiece-prepared canals, the amount of extruded debris was significantly lower in premolar teeth.

## Introduction

Several factors are involved in induction of post endodontic flare-ups including inadequate debridement, debris extrusion (DE), single-visit treatment, preparation beyond the apex of the root, retreatment cases and existence of periapical lesions [1, 2]. Healing process after endodontic treatment also depends on several factors with the amount of DE into the periapical area being the most important one [3].

DE can play an important role in increasing the inflammatory response in the periradicular area [4, 5] that could also delay the healing of periapical lesion [6, 7]. Thus reducing the amount of extruded debris during endodontic treatment is proposed as a method of preventing inter-appointment and post-treatment pain and flare-up [8, 9].

Nickel-titanium (NiTi) hand files are 2-3 times more elastic than stainless steel files due to their very low modulus of elasticity (MOE). Also because of the ductility, NiTi files have shown higher resistance to torsional fracture [10]. According to the structural characteristics of these devices, their use is likely to reduce the extrusion of debris from the apical end.

It is stated that during manual instrumentation, the force exerted on the file may push the preparation debris beyond the apex. Carrying files by hand for several times is often tedious and exhausting both for the dentist and the patient. On the other hand, by reciprocating back-and-forth file motion, less debris will be packed through the apex compared to the up and down filing motion which is due to the Archimedes’ screw effect [11-13]. Reciprocation also lowers the risk of file fracture [12, 14, 15]. 

Handpieces capable of carrying hand files into the canal with back-and-forth motion (reciprocation) were first designed aiming at simplifying root canal preparation. Endolift (Kerr, Karlsruhe, Germany) was first introduced in 1982 to shorten the operation time and reduce operator’s fatigue. The primary handpiece had a 90^°^ reciprocal oscillating motion. Then it was followed by M4 Safety Handpiece that offered a 30^°^ reciprocating movement [16]. Some studies have shown that patient’s pain and inflammation during and after the instrumentation with reciprocation handpieces may reduce due to the high velocity and harmony of the motions. Moreover, the risk of file anchoring in canal or screwing effect is reduced which is common in full rotary motions [17].

Since one of the most important causes of post-endodontic flare-ups is DE, the purpose of this study was to compare the amount of DE in straight and curved root canals after preparation with NiTi hand files either installed on reciprocating handpieces or used manually.

## Materials and Methods

A total number of 40 samples were chosen for this study [8] including mesiobuccal (MB) roots of 20 extracted human maxillary first molars with radius of curvature between 10 to 20 degrees, (specified according to the Schneider’s method [18]) and roots of 20 single-canal mandibular premolars (20-30 degrees curvature) were selected. Periapical radiographs were taken and teeth with calcification, open apices, internal or external root resorption, severe curvature and cracked root, were excluded. Afterwards the root surfaces were mechanically cleaned of calculus and soft tissues and disinfected in 0.5% sodium hypochlorite (NaOCl) solution for 24 h and stored in 0.1% solution of distilled water and Timol. Then the crown of the teeth were cut with a diamond disk so that all samples had 19 mm root lengths. Apical patency was controlled with a #10 K-file (Mani, Tochigi, Japan). Then, all roots were instrumented with a #15 NiTi hand file (NiTi flex, Maillefer, Ballaigues, Switzerland). The samples were randomly divided into two experimental groups (A and B) (*n*=20) including two subgroups each with 10 mandibular premolars (A1 and B1) and 10 MB roots of maxillary first molars (A2 and B2). In group A, samples were prepared using NiTi hand files installed on a reciprocal handpiece (NSK, TEP-E10R, Nakanishi Inc., Tokyo, Japan) [19]. In group B, canals were prepared with conventional step-back technique using similar files.

Collection of apically extruded debris was conducted according to the technique developed by Fairbourn *et al*. [20] and modified by Myers and Montgomery [10]. Briefly, each root was forced into a pre-cut rubber stopper and placed into a plastic vial which was mounted into a glass flask. Because of balancing between the air pressure inside and outside the vials, a bent 25-gauge needle (Supa, Tehran, Iran) was also forced alongside the stopper to use as a drainage cannula. In both groups the size of apical preparation was set at #35 and #40 for the MB and premolar roots, respectively. Each file was replaced after 6 times of usage. Two mL of distilled water was used for irrigation of the root canals using insulin syringes that entered the middle third of the canals. 

After completeness of the preparation, the vials were placed in an incubator with temperature of 60^º^C for 72 h. Two vials of distilled water were applied as the control groups. Then to avoid the interference of humidity, they were located in desiccators. Before weighing the debris, empty vials were weighed with an electronic semimicro balance (Sartorius AG, Göttingen, Germany). Finally, by using SPSS software (SPSS version 21.0, SPSS, Chicago, IL, USA) the mean dry weights of debris were compared regardless of tooth type, using the two independent samples t-test. Furthermore, two-way ANOVA was applied for analyzing the subgroups, and if the interaction between instrumentation technique and tooth type was significant, the two independent samples t-test was performed separately on each tooth type. The level of significance was set at 0.05.

## Results

Apical DE in groups A and B was 0.505±0.176 and 1.245±0.863 mg, respectively (Table 1). According to the results of t-test, regardless of the tooth type, the mean weight of extruded debris was significantly lower in group A (*P<*0.0001). 

In addition, since the assumptions of two-way ANOVA did not imply for debris weight, transformed natural logarithm form was used. 

**Table 1:** Mean (SD) of debris extrusion (in mg)

**Instrumentation technique**	**Tooth type (N)**	**Mean (SD) **
**Hand instrumentation **	**Premolar (10)**	1.57 (0.970)
	**Molar (10)**	0.92 (0.630)
	**Total (20)**	1.245 (0.863)
**Reciprocating handpiece **	**Premolar (10)**	0.41 (0.199)
	**Molar (10)**	0.60 (0.169)
	**Total (20)**	0.505 (0.176)

**Figure 1:**
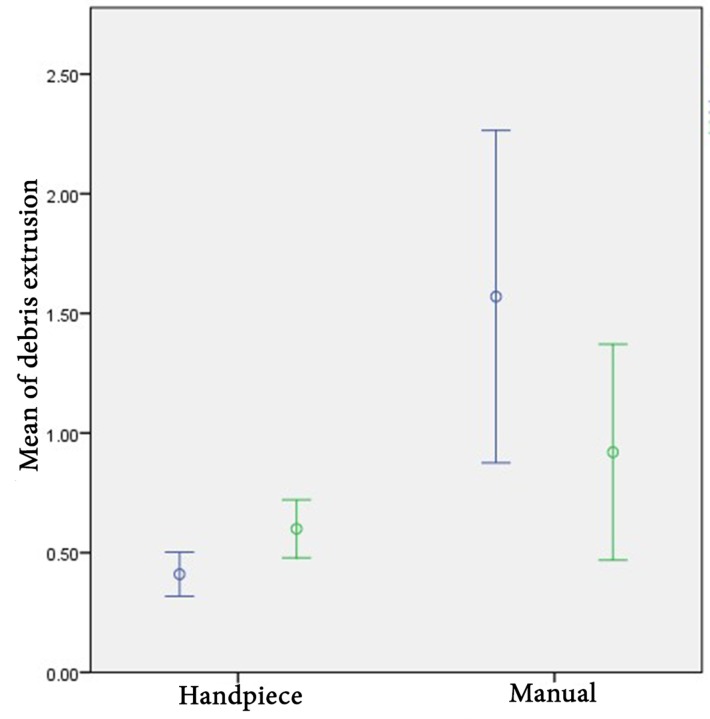
Apical extrusion of debris (mg) in subgroups

The interaction between instrumentation method and tooth type was significant (*P*=0.005); meaning that for evaluating the effect of instrumentation method on the amount of DE, separate independent samples t-tests was performed on premolar and molar teeth. Reciprocating handpiece resulted in significantly lower amount of DE in premolars (*P*<0.001), while there was not a significant difference between the amount of DE in molars in each study group (*P*=0.20) (Figure 1). The same results were obtained for the debris weight.

## Discussion

This *in vitro* study compared the amount of apical DE after root canal preparation in MB roots of maxillary molars and mandibular premolars using NiTi hand files either manually or installed on 60^°^ reciprocating air-driven handpiece. The mean weight of extruded debris was significantly higher in manually prepared teeth.

One of the major problems in Endodontics is the apical DE [21]. This phenomenon happens in all the instrumentation techniques to different extends [22-25]. Wise choice of preparation techniques that pushes less debris from the apex is helpful [26]. In our study, the significant difference in amount of DE was demonstrated between reciprocating air-driven system and manual step-back technique using hand NiTi files. It was also shown that the DE diminished following the use of reciprocating air-driven and balanced-force technique; this is consistent with the results of a recent investigation showing the absence of significant differences between DE after canal preparation with NiTi rotary instruments and the Endolift system. Also the amount of DE in manual step-back technique using K-files was more than both mentioned techniques [27]. 

It is demonstrated that root canal preparation by manual step-back technique causes more extrusion of debris than rotary systems such as FlexMaster, RaCe and ProFile [21, 26, 28]. Some other investigations revealed that the amount of DE in manual step-back technique was significantly more than the balanced-force and the crown-down preparation techniques; no significant difference existed between the balanced-force and the rotary instrumentation, either [22, 29, 30]. 

In the current study, samples were precisely included according to the inclusion criteria and all the steps of the study were performed by one trained examiner. The apical patency can potentially lead to inappropriate length control and more DE during the root canal instrumentation [29]. In addition, to have an easy and reliable reference point for measuring the working length, the crowns were cut with a diamond disc, and root length remained at the similar length of 19 mm [29]; thus, the differences in the amounts of DE could be attributed to the various instrumentation techniques and not due to the tooth morphology. Furthermore, since debris can be pushed into the periradicular tissues during root canal irrigations [31], in the present study irrigation was done passively.

However, since there was no similarity of the samples between the present and previous studies, the results could not be compared with the last ones. Moreover, in clinical situations the pressure of periapical tissues may act as a natural barrier against extrusion of the debris and thus different results can be obtained. In future studies, the amount of required pressure that can resist DE should be determined.

## Conclusion

According to the results of this study, using reciprocating air-driven handpieces with NiTi hand files can lead to significantly lower debris extrusion in comparison with the manual step-back technique.
